# Analytical Methods for Determining Third and Fourth Generation Fluoroquinolones: A Review

**DOI:** 10.1007/s10337-016-3224-8

**Published:** 2016-12-23

**Authors:** Andrzej Czyrski

**Affiliations:** The Department of Physical Pharmacy and Pharmacokinetics, Poznań University of Medical Sciences, 6 Swiecickego Street, 60-781 Poznan, Poland

**Keywords:** HPLC, UV/Vis spectroscopy, Fluorescence spectroscopy, Mass spectrometry, Pharmacokinetics, Clinical pharmacokinetics

## Abstract

**Abstract:**

Fluoroquinolones of the third and fourth generation posses wide bactericidal activity. Monitoring concentrations of antibacterial agents provides effective therapy and prevents the increase of bacterial resistance to antibiotics. The pharmacodynamic parameters that best describe fluoroquinalone activity are AUC/MIC and *C*_max_/MIC. Determining the level of this type of drug is essential to reach the effective concentration that inhibits the growth of bacteria. Determining the pharmaceutical formulation confirms the purity of a substance. Many methods have been developed to determine the level of these substances. They involve mainly the following analytical techniques: chromatography, capillary electrophoresis, and spectroscopy. The separation techniques were combined with different measuring devices, such as ultraviolet (UV), fluorescence detector (FLD), diode array detector (DAD), and mass spectrometry (MS). The analytical procedures require proper sample pre-conditioning such as protein precipitation, extraction techniques, filtration, or dilution. This paper reviews the reported analytical methods for the determining representatives of the third and fourth generation of fluoroquinolones. Attention was paid to pre-conditioning of the samples and the applied mobile phase. This report might be helpful in the selection of the proper procedure in determining the abovementioned drugs in different matrices.

**Graphical abstract:**

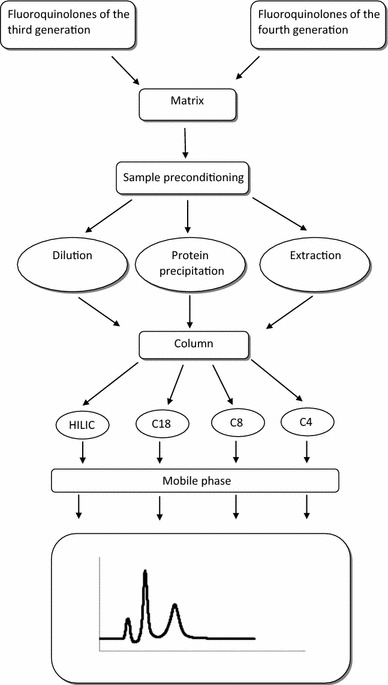

## Introduction

Fluoroquinolones are a vast class of synthetic bactericidal agents widely used in treatment. In 1963 nalidixic acid was the first quinolone approved by FDA. The intensive development of this group of antibacterial drugs was in the 1980s with the discovery that a combination of fluorine atoms at position 6 and a piperazinyl group at position 7 of the quinoline ring expands the spectrum of bactericidal activity. This modification in structure produced norfloxacin, the first of a new generation of fluoroquinolones [1]. They are divided into four generations. The adjustment of the drug to a proper class is based on its pharmacological activity. Fluoroquinolones comprise a broad spectrum of activity against Gram-positive, Gram-negative, and atypical bacteria, as well as *Mycoplasma*, *Chlamydia,* and *Legionella*. Their activity is based on inhibition of bacterial enzymes: DNA gyrase and DNA topoisomerase IV. These enzymes are necessary to separate bacterial DNA. This activity leads to inhibition of cell replication [2–6].

The activity of fluoroquinolones strongly depends on their concentration. Pharmacokinetic parameters may exhibit interpatient variability, especially in some groups of patients, such as the critically ill, those with renal impairment, or hospitalized patients. Pharmacodynamic parameters that best describe the efficacy are the area under the plasma concentration–time curve to minimum inhibitory concentration (AUC/MIC) and maximum plasma drug concentration to minimum inhibitory concentration (*C*_max_/MIC). The optimal value of these parameters provides the effective pharmacotherapy of bacterial diseases and thus prevents bacterial resistance and lack of therapy efficacy [7–11].

In the analysis of fluoroquinolones many high performance liquid chromatography (HPLC) methods with different detection techniques were applied. The most common is HPLC with ultraviolet (HPLC-UV) or fluorescence detection (HPLC-FLD). Another detector combined with the HPLC system is mass spectrometry (MS) (HPLC–MS). These methods are common for determining drugs in serum; however, mass detection makes it possible to determine very low concentration in matrix. Another separation technique for analysis of the fluoroquinolone levels is capillary electrophoresis (CE) combined with UV or FLD. The determination of drug level in pharmaceutical formulation can be performed by both HPLC techniques and others such as UV-spectroscopy, voltamperometry, or even nuclear magnetic resonance (NMR). All the aforementioned methods require proper preparation of the sample. The pharmaceutical formulations are the least complex matrices—only dilution is required. Physiological fluids (blood, bile, saliva, and urine) and tissues homogenates require a more complex technique of separation. This is due to the presence of endogenous substances that may appear on the chromatogram or electropherogram during analysis. The key factor is to optimize the conditions of the analysis (use of the proper solvent or buffer) and the sample preparation. In this case the sample preparation may involve dilution which might be applied for urine, protein precipitation or extraction applied in more complex matrices (blood, serum, tissue homogenates). This paper reports information about the methods for determining representatives of the third and fourth generation of fluoroquinolones in different matrices. The methods are divided according to the used analytical technique used and preconditioning of samples for analysis.

## Third Generation Fluoroquinolones

The third generation representatives are levofloxacin (LEVO), balofloxacin (BALO), pazufloxacin (PAZU), and sparfloxacin (SPAR) [12]. LEVO is used in the treatment of the community-acquired pneumonia (CAP), acute maxillary sinusitis, and acute exacerbation of chronic bronchitis. LEVO is also used in the eradication of *Helicobacter pylori* when standard therapies fail. The oral and intravenous administration of LEVO are equivalent due to its full bioavailability. It is poorly metabolized—after 48 h about 87% of unchanged drug is eliminated in urine. The main metabolites are *N*-oxide and desmethyllevofloxacin, and they are inactive [12–14]. BALO exhibits excellent antibacterial activity against Gram-positive bacteria such as multi-drug-resistant *Staphylococci* and *Pneumococci*. It is metabolized in the kidneys to glucuronide and *N*-desmethyl derivative [15]. PAZU has strong activity against Gram-negative bacteria, and it easily permeates the liver tissue, gallbladder tissue, and bile. This indicates that PAZU might be useful in the treatment of patients with the liver disease [16]. SPAR is reported to be more active in vitro than ciprofloxacin against *Mycobacteria* and Gram-positive bacteria including *Streptococcus pneumoniae* and other *Streptococci* and *Staphylococci* [17].

### Levofloxacin

There are many analytical techniques for quantitative analysis of this drug in different matrices (Table 1). Most of them are based on reversed phase HPLC. These techniques are well suited due to the solubility of LEVO in water. The most common applied detectors are UV [18–25] and FLD [20, 26–31]. However, if the lower level of quantification is required, mass detection (MS) can be applied [32–35]. In MS/MS analysis the following multiple reaction modes (MRM) are employed: *m*/*z* 362.7 → 261.2 [32], *m*/*z* 362.1 → 318.1 [34], and *m*/*z* 362.2 → 261.2 [35]. For single MS the following selected reaction monitoring is observed *m*/*z* 362 → 318 [33]. The other detector that can be applied is photodiode array detector PDA [36]. The mobile phase is a mixture of water or aqueous buffer and organic solvent. Triethylamine (TEA) is used as an addition to mobile phase. TEA is an ion pair reagent added to water that improves the shape of the peak. Its content does not exceed 1%, and the pH of the mobile phase is slightly acidic [19, 21, 25, 26, 31, 36]. The proper pH value is shifted with orthophosphoric acid. The addition of ion pair reagent improves the quality of the separation due to the presence of the negatively charged carboxyl group. The other polar constituent might be the phosphate buffer consisting of either sodium or potassium phosphates in the following range of concentrations 10–30 mM [19, 24, 29, 36]. The most common organic solvent in HPLC separation is acetonitrile (ACN) [20–22, 25–27, 29–31, 33, 35, 36]. Its content is within the range 14–43% for isocratic elution [20–22, 25, 26, 31, 36] and is also applied in gradient elution; however, in this case the content of ACN varies in time [27, 33, 35]. The high content of ACN is characteristic for separation on a hydrophilic interaction liquid chromatography (HILIC) column where the content of organic solvent is higher than 80% [32].

**Table 1:** The methods for determining the fluoroquinolones in different matrices

Analysed compound	Sample	Method	Sample preparation	LOD/LOQ	References
LEVO	Pure form, pharmaceutical formulation	VIS-spectroscopy, *λ* = 424 nm for LEVO-BPB and *λ* = 428 nm for LEVO-BCG	LLE with chloroform	LLOQ for LEVO-BCG and LEVO-BPB—1.85 µg mL^−1^	[41]
LEVO	Pharmaceutical formulation	HPLC-UV, chiral separation with mixture of l-leucine and Cu^2+^ ions (CMPA), column: C18 OptimaPak 150 mm × 4.6 mm, 5 μm), mobile phase: CMPA solution—MeOH (88:12), isocratic elution	Not reported	LLOQ—0.5 mg L^−1^	[18]
LEVO	Urine	Adsorptive square-wave anodic stripping voltammetry	Dilution with acetate buffer	LOD—10 μg mL^−1^	[44]
LEVO	Sputum	MEPS–HPLC–PDA, column: C8 discovery column (250 mm × 4.6 mm, 5 μm), mobile phase: phosphate buffer (30 mM, pH 2.5, 1% TEA) and ACN (1% TEA) 86:14 (v/v) isocratic elution	MEPS extraction	LOD—0.017 μg mL^−1^ LOQ—0.05 μg mL^−1^	[36]
LEVO	Tablets, urine and serum	Fluorescent spectroscopy, *λ*_excitation_ = 292 nm, *λ*_emission_ = 494 nm, the fluorescence was enhanced by micelle	Tablets were powdered and dissolved in an ethanol–water mixture; urine was dissolved in water; serum did not require pre-treatment	LOD—10 ng mL^−1^ LOQ—30 ng mL^−1^	[43]
LEVO	Pharmaceutical formulation, urine, plasma	^1^H NMR spectroscopy	Tablets were powdered and dissolved in borate buffer; urine and plasma samples were diluted with D_2_O	Pharmaceutical formulation: LOD—0.134 mg·0.6 mL^−1^, LOQ—0.446 mg·0.6 mL^−1^; Urine: LOD 0.015 mg·0.6 mL^−1^, LOQ—0.05 mg·0.6 mL^−1^; Plasma: LOD—0.153 mg·0.6 mL^−1^, LOQ—0.5 mg·0.6 mL^−1^	[45]
LEVO	Pharmaceutical formulation	Synchronous scanning room temperature phosphorimetry	Boiling in a Soxhlet apparatus	ALOD—13 ng, ALOQ—41 ng	[46]
LEVO	Plasma	HILIC–MS/MS, column: HILIC Silica Column (50 mm × 3.0 mm, 5 μm), mobile phase: ACN—ammonium formate (100 mM pH 6.5) 82:18 (v/v), isocratic elution, temperature 30 °C	LLE with dichloromethane	LLOQ—10 ng mL^−1^	[32]
LEVO	Tissue homogenate	LC–MS, column: Symmetry C18 (100 mm × 2.1 mm, 3.5 μm), mobile phase: A:ACN, water, formic acid (3:97:0.2 v/v/v), B: ACN: formic acid (100:0.2 v/v), gradient elution	SPE	LOQ—0.02 μg g^−1^	[33]
LEVO	Reaction mixture	RP–HPLC–UV, column: ACE C18 column (250 mm × 4.6 mm, 5 μm), mobile phase: A: 25 mM NaH_2_PO_4_, 0.5% TEA pH 6.0, B: methanol, gradient elution, temperature 40 °C	Dilution of the samples with a mixture of water:ACN (60:40)	Not reported	[19]
LEVO	Plasma, bone tissue, BAL	HPLC–UV, column: ABZ + Supelcosil (150 mm × 4.6 mm, 5 μm), mobile phase: 0.4% TEA pH 3.0: ACN (83:17 v/v), isocratic elution	SPE	LOD—0.05 μg g^−1^ (plasma), 0.1 μg/g (BAL), 0.2 μg g^−1^ (bone), LOQ—0.2 μg g^−1^ (plasma), 0.4 μg g^−1^ (BAL), 0.5 μg g^−1^ (bone)	[21]
LEVO	Tissues, plasma	HPLC–MS/MS, column: C4 Welsch Materials (250 mm × 4.6 mm, 5 μm), mobile phase: A: 0.05% formic acid, B: methanol, gradient elution	Protein precipitation with methanol	LOD—0.05 μg g^−1^ (tissues), 6.6 ng mL^−1^ (plasma), LLOQ—0.13 μg g^−1^ (tissues), 21.8 ng mL^−1^ (plasma)	[34]
LEVO	Bulk and marketed formulations	UV spectroscopy, *λ* = 292 nm	Sample dilution	LOD—0.021 μg mL^−1^, LOQ—0.064 μg·mL^−1^	[42]
LEVO	Tablets	HPLC–UV, column: C18 Cosmosil MS II, mobile phase: 0.05 M citric acid monohydrate: 1 M ammonium acetate: ACN (84:1:15), isocratic elution	Tablets were pulverized, dissolved, and diluted	Not reported	[22]
LEVO	Plasma, dialysate	HPLC-FLD, column: YMC PRO C18 (150 mm × 2 mm, 5 μm), mobile phase: A: (MeOH: 1.0 M NH_4_OAc:H_2_O—10:5:85 v/v/v), B: (MeOH: 1.0 M NH_4_OAc:H_2_O—40:5:55 v/v/v), gradient elution	Protein precipitation with 50% TFA	LLOQ—0.1 μg mL^−1^	[28]
LEVO	Plasma, urine	HPLC–UV, column: C18 Intersil ODS-2 (250 mm × 4.6 mm, 5 μm), mobile phase: (5 mM CuSO_4_, 10 mM l-isoleucine):methanol 87.5:12.5, isocratic elution, temperature 35 °C	LLE with dichloromethane	LLOQ—0.08 μg mL^−1^ (plasma), 23 μg mL^−1^ (urine)	[23]
LEVO	Blood, bile	HPLC-FLD, column:C18 LiChrospher (250 mm × 4.6 mm, 5 μm), mobile phase: ACN: 1 mM 1-octanesulfonic acid (40:60 v/v, pH 3.0), isocratic elution	Microdialysis	LOD—50 ng mL^−1^, LLOQ—0.1 μg mL^−1^	[30]
LEVO	Plasma	HPLC-FLD, column: C18 LiChroCART (125 mm × 4 mm), mobile phase: ACN—0.4% TEA pH 3.0 (24:76 v/v), isocratic elution	Protein precipitation with ACN	LOD 0.03 mg L^−1^, LLOQ 0.15 mg L^−1^	[31]
LEVO	Urine	CE with electroluminescence detection, capillary: a fused-silica capillary 55 cm × 50 μm i.d. × 375 μm o.d., 18 kV, buffer: 20 mM PBS pH 8.0	Precipitation with ACN	LOD—6.4 × 10^−7^ M, LOQ—1.4 × 10^−6^ M	[38]
LEVO	Water	CE with UV detection, capillary: fused silica capillary 80 cm × 75 μm i.d., BGE (3 M acetic acid, 49 mM ammonium acetate in 55:45 v/v methanol: ACN), 30 kV, temperature 20 °C	Dispersive liquid–liquid microextraction	LOD—5.74 μg mL^−1^, LOQ—19.1 μg mL^−1^	[40]
LEVO/PAZU	Serum	HPLC-FLD, column: Intersil C8-3 (250 mm × 4.6 mm, 5 μm), mobile phase: 1% TEA pH 3.0: ACN (86:14 v/v), isocratic elution	Protein precipitation with 6% HClO_4_ and methanol	LLOQ—0.1 μg mL^−1^ (LEVO and PAZU)	[26]
LEVO/MOXI/GATI	Tablets	CE with UV detection, capillary: a fused-silica capillary 48.5 cm × 50 μm i.d. × 375 μm o.d., 25 kV, buffer: 25 mM TRIS/hydrochloride and 15 mM sodium tetraborate pH 8.87, temperature 25 °C	Tablets were powdered and dissolved	LOD—1.02 mg L^−1^, LOQ—3.40 mg L^−1^ (LEVO), LOD—1.53 mg L^−1^, LOQ—5.11 mg L^−1^ (MOXI), LOD—1.51 mg L^−1^, LOQ—5.03 mg L^−1^ (GATI)	[39]
LEVO/MOXI/GATI/TROVA	Plasma	HPLC–UV/FLD, column: Adsorbsphere C18 (250 mm × 4.6 mm, 5 μm), mobile phase: A: 10 mM SDS, 10 mM TBAA, 25 mM citric acid, B: ACN pH 3.5 (57:43 v/v), isocratic elution	Ultrafiltation of pre-conditioned sample with SDS 0.5% solution	LLOQ—50 ng mL^−1^ for UV detection, and 20 ng mL^−1^ for FLD detection (LEVO, MOXI, GATI, TROVA)	[20]
LEVO/MOXI/PAZU/GATI//TROVA	Plasma	HPLC-FLD, column: C18 LiChroCART Purospher Star (55 mm × 4.0 mm, 3 μm), mobile phase: A: 0.1% formic acid adjusted to pH 3.0 with TEA, B: ACN and C: MeOH, gradient elution	Protein precipitation with ACN	LOD—0.01 µg mL^−1^ (LEVO, PAZU, MOXI), 0.02 µg mL^−1^ (TROVA), 0.0025 µg mL^−1^ (GATI); LOQ—0.02 µg mL^−1^ (LEVO, PAZU, MOXI), 0.04 µg mL^−1^ (TROVA), 0.005 µg mL^−1^ (GATI)	[27]
LEVO/MOXI/GATI	Serum	HPLC-FLD with column switching, ABZ + Plus Supelcosil (150 mm × 4.6 mm, 5 μm), mobile phase: I. (10 mM K_2_HPO_4_ pH 5.4: ACN 98:2 v/v), II. (10 mM KH_2_PO_4_ pH 2.5 with 2 mM TBAmBr: ACN 88:12 v/v), isocratic elution	Online extraction on pre-column	LOD—60 ng mL^−1^, LOQ—125 ng mL^−1^ (LEVO) LOD—35 ng mL^−1^, 125 ng mL^−1^ (MOXI), LOD—120 ng mL^−1^, LOQ—162.5 ng mL^−1^ (GATI)	[29]
LEVO/MOXI/GATI	Plasma	HPLC–UV, column: Hypersil BDS C18 column (250 mm × 4.6 mm, 5 μm), mobile phase: 20 mM NaH_2_PO_4_ pH 3.2: ACN (75:25 v/v), isocratic elution	Protein precipitation with ACN:methanol mixture (1:1)	LLOQ—0.1 µg mL^−1^ (LEVO, MOXI and GATI)	[24]
LEVO/GATI	Pharmaceutical formulations	HPLC–UV, column: C18 LiChrospher 100 (125 mm × 4.0 mm, 5 μm), mobile phase: water and ACN (80:20 v/v) with addition of 0.3% TEA, pH 3.3, isocratic elution	Tablets were powdered and dissolved and ampoules were diluted	LLOQ—4 μg mL^−1^	[25]
LEVO/SPAR	Chicken breast muscle	HPLC–MS/MS, column: C18 Symmetry (150 mm × 4.6 mm, 5 μm), mobile phase: A:54 mM formic acid 10 mM CH_3_COONH_4_, B: ACN, gradient elution, temperature 30 °C	Extraction with ACN with 0.3% addition of phosphoric acid (v/v)/water (70:30) and hexane	LOD—3.6 ng g^−1^, LLOQ—11.9 ng g^−1^ (LEVO) LOD—2.7 ng g^−1^, LLOQ—8.9 ng g^−1^ (SPAR),	[35]
BALO	Plasma	HPLC–MS, column: C18 Agilent ZORBAX 300SB (150 mm × 2.1 mm), mobile phase: methanol: water (10 mM CH_3_COONH_4_, pH 3.0) (40:60 v/v), isocratic elution, temperature 40 °C	LLE with the mixture dichloromethane and ethyl acetate (20:80 v/v)	LOD—0.02 µg mL^−1^, LLOQ—0.03 µg mL^−1^	[15]
BALO	Tablets	HPLC–UV, column: C18 Zodiac (150 mm × 4.6 mm, 5 μm), mobile phase: 0.01 M KH_2_PO_4_: ACN pH 6.5, (40:60 v/v), isocratic elution, temperature 30 °C	Sample dilution	LOD—0.85 µg mL^−1^, LOQ—2.58 µg mL^−1^	[48]
PAZU	Saliva, gingival crevicular fluid, serum	HPLC–UV, C18 Agilent ZORBAX SB (250 mm × 4.6 mm, 5 μm), mobile phase: ACN: 0.5% phosphoric acid containing 1% TEA (155:850 v/v), isocratic elution	Protein precipitation with methanol	LOD—10 ng mL^−1^	[49]
PAZU	Tablets, milk	CE with potential gradient detection, capillary: fused-silica capillary (8.5 cm × 75 μm i.d. × 375 μm o.d., 3 kV, buffer: 30 mM Tris and 4 mM phosphoric acid at pH 8.9, temperature 20 °C	LLE with dichloromethane	LOD—39 ng mL^−1^, LOQ—130 ng mL^−1^	[50]
PAZU	Muscle	CE with potential gradient detection, capillary: fused-silica capillary (7.8 cm × 50 μm i.d., 3 kV, buffer: 30 mM Tris and 3 mM phosphoric acid at pH 9.0, temperature: ambient	LLE with dichloromethane	LOD—0.3 mg L^−1^	[51]
PAZU/GATI	Urine	CE with UV detection, capillary: fused-silica capillary (50 cm × 50 μm i.d. × 375 μm o.d., 10 kV, buffer: 70 mM phosphate buffer and 40 mM hydroxypropyl β-cyclodextrin at pH 5.04 for pazufloxacin and 20 mM hydroxypropyl β-cyclodextrin at pH 3.90 for gatifloxacin, temperature: 20 °C	Dilution with water	LOD—7 µg mL^−1^ for PAZU	[52]
SPAR	Pharmaceutical formulations	UPLC-UV, column: C18 Waters Acquity HSS T-3 column (100 mm × 2.1 mm, 1.8 μm), mobile phase: A: 0.1% orthophosphoric acid, B: ACN, gradient elution, temperature 50 °C	Tablets were pulverized and dissolved, eye drops were dissolved	LOD—0.2 µg mL^−1^, LOQ—0.6 µg mL^−1^	[17]
SPAR	Plasma, urine	HPLC–UV, column: C18 YMC pack A-312 (150 mm × 6 mm, 5 μm), mobile phase: 5% acetic acid:ACN: methanol (76:12:12) with 1% addition of TEA to mobile phase, isocratic elution, temperature 50 °C	Protein precipitation with 20% perchloric acid for plasma samples. The urine samples were diluted with water	LOQ—0.025 mg·L^−1^ (plasma), 0.5 mg·L^−1^ (urine)	[53]
SPAR/MOXI/GATI	Plasma	HPLC–UV, column: C18 Kromasil 100 (250 mm × 4.6 mm, 5 μm), mobile phase: NaH_2_PO_4_ (pH 2.5): ACN (80:20 v/v), isocratic elution, temperature 35 °C	LLE with ethyl acetate	LOQ—100 ng·mL^−1^ for each compound	[54]
SPAR	Plasma	HPLC–MS/MS, column: C18 Atlantis (50 mm × 2.1 mm, 3 μm), mobile phase: 10 mM CH_3_COONH_4_ (pH 4.0): ACN (20:80 v/v), isocratic elution	Protein precipitation with ACN	LLOQ—10 ng·mL^−1^	[55]
SPAR/PAZU/GATI	Serum	CE with UV detection, capillary: fused-silica capillary (65 cm × 50 μm i.d. × 375 μm o.d., 10 kV, buffer: 12 mM disodium tetraborate pH 9.08 in addition of silica nanoparticles (5.2 µg/mL), temperature: 25 °C	Dilution with buffer solution	LOD—2 µg mL^−1^ (for GATI, PAZU), 2.5 µg mL^−1^ (SPAR), LLOQ—5 µg mL^−1^ (for GATI, SPAR), 6 µg mL^−1^ (PAZU)	[56]
MOXI	Pure substance, tablets	Vis-spectroscopy, *λ* = 623 nm (complexes with MBTH in the presence with Ce IV ions), *λ*_max_ = 623 nm	Tablets were pulverized and dissolved	LOD—0.043 μg mL^−1^, LOQ—1.89 μg mL^−1^	[85]
MOXI	Eye drops	HPLC–UV–DAD, column: C8 BDS Hypersil column (250 mm × 4.6 mm, 5 µm), mobile phase: 20 mM phosphate buffer with 0.1% TEA pH 2.8: methanol (38.5:61.5 v/v), isocratic elution	Eye drops were diluted with mobile phase	LOD—0.316 μg mL^−1^, LOQ—1.014 μg mL^−1^	[66]
MOXI	Plasma	LC–MS/MS, column: ODS C18 (150 mm × 4.6 mm, 5 µm); mobile phase: methanol: 0.03% TEA (85:15, v/v), isocratic elution, temperature 30 °C	Protein precipitation with methanol	LLOQ—0.1 μg mL^−1^	[80]
MOXI	Eye drops	RP–HPLC–UV, column: Grace Smart C18 (250 mm × 4.6 mm, 5 µm), mobile phase: methanol: 25 mM KH_2_PO_4_ with addition of 0.5% TEA pH 3.2 (60:40 v/v), isocratic elution	Eye drops diluted with mobile phase	LOD—0.098 μg mL^−1^, LOQ—0.327 μg mL^−1^	[74]
MOXI	Saliva	HPLC-FLD, column: C18 Lichrospher 100 RP-18e (150 mm × 4.6 mm, 5 µm), mobile phase: 50 mM phosphate buffer (KH_2_PO_4_) pH 2.6: ACN (80:20 v/v), isocratic elution	Protein precipitation with 7% HClO_4_	LOD—0.03 μg mL^−1^, LOQ—0.1 μg mL^−1^	[76]
MOXI	Blood	LC–MS/MS, column: C18 Hy-Purity (50 mm × 2.1 mm, 5 µm), mobile phase: A: (ammomium acetate 10 g/L, acetic acid 35 mg/L, trifluoroacetic anhydride 2 mL/L water), B: water, C: ACN, gradient elution	Extraction with cyanoimipramine in mixture of methanol and water	LLOQ—0.05 mg L^−1^	[81]
MOXI	Pharmaceutical formulations	HPLC–UV, column: Waters Xterra C18 Purity (50 mm × 2.1 mm, 5 µm), mobile phase 2% TEA pH 6.0 and ACN (90:10 v/v), isocratic elution	Formulations were powdered and dissolved	LOD—0.05 μg mL^−1^, LOQ—0.2 μg mL^−1^	[60]
MOXI	Plasma	HPLC–UV, column: C8 Kromasil (250 mm × 4.6 mm, 5 µm), mobile phase: ACN: methanol: (20 mM, 1% TEA pH 3.0) KH_2_PO_4_ (15:20:65 v/v/v) at 30 °C isocratic elution	LLE with dichloromethane	LOD—0.015 μg mL^−1^, LLOQ—0.05 μg mL^−1^	[75]
MOXI	Plasma	LC ESI–MS/MS, column: BDS Hypersil C18 (100 mm × 4.6 mm, 5 µm), mobile phase: 0.1% formic acid and ACN (60:40 v/v)	SPE	LOD—50 pg mL^−1^, LOQ—1 ng mL^−1^	[82]
MOXI	Vitreous and aqeous humor	HPLC–FLD, ACCQ Taq aminoacid analysis column (150 mm × 3.9 mm, 4 µm) mobile phase, A: ACN, methanol, 0.05 M TBA·Cl, TFA (37.5:12.5:949:1 v/v/v/v), B: ACN: methanol, 0.05 M TBA·Cl, TFA (75:25:899:1v/v/v/v) pH 3.0, gradient elution, temperature 30 °C	Dilution with mobile phase for aqueous humor and protein precipitation with ACN for vitreous humor	LOD—10 ng mL^−1^	[77]
MOXI	Plasma	HPLC–FLD, column: supelco LC-Hisep column (150 mm × 4.6 mm, 5 µm), mobile phase: ACN: 0.25 M Na_3_PO_4_ pH 3.0 (5:95 v/v), isocratic elution	Microfiltration in syringe	Plasma: LOD—1 μg L^−1^, LOQ—3 μg L^−1^, Water: LOD—0.1 μg L^−1^, LOQ—1 μg L^−1^	[78]
MOXI/SPAR	Plasma	HPLC–DAD/FLD, column: Altantis dC18 column (150 mm × 4.6 mm, 5 μm), mobile phase: ACN and 0.1% TFA, gradient elution	Protein precipitation with ACN	LOQ—0.04 μg mL^−1^ (MOXI) FLD detection, 0.3 μg mL^−1^ (SPAR) DAD detection	[57]
MOXI/GATI	Muscle	CE with capacitively coupled contactless conductivity detection, capillary: fused-silica capillary (42 cm × 50 μm i.d. × 375 μm o.d.) 13 kV, buffer: BGE 85% (10 mM tartaric acid, 14 mM sodium acetate) and 15% methanol (v/v) pH 3.8, room temperature	LLE with dichloromethane, the homogenate was defatted with n-hexane	LOD—0.33 μg mL^−1^ (MOXI), 0.45 μg mL^−1^ (GATI), LOQ—1.4 μg mL^−1^ (MOXI), 2.1 μg mL^−1^ (GATI)	[83]
MOXI	Drug substance and ophthalmic drug	CE–DAD, capillary (40 cm × 50 μm i.d.) 13 kV, buffer: BGE (12.5 mM TEA pH 2.5 with 5% highly sulfated γ-cyclodextrin and 6% ACN), temperature 20 °C	Sample dilution	LOQ—0.055 μg mL^−1^	[84]
MOXI/GATI	Aqueous humor and vitreous humor	HPLC-PAD/FLD, column: LiChrospher RP-18 (125 mm × 4 mm, 5 µm), mobile phase: ACN—0.1% TFA (pH 3.0) with 30 mM TBA·Cl (20:80), isocratic elution	Aqueous humor samples were diluted, vitreous humor samples were extracted by SPE	LLOQ—0.01 μg mL^−1^ (MOXI and GATI)	[79]
TROVA	Plasma	HPLC–UV, column: C18 Phenomenex Luna (150 mm × 4.6 mm, 5 µm), mobile phase: A: 17.5 mM NaH_2_PO_4_ 1.5 mM tetrabutylammonium hydroxide pH 3.0, B: ACN and methanol (1:1 v/v), gradient elution	Protein precipitation with 20% HClO_4_	LOD—2 ng mL^−1^ LLOQ—10 ng mL^−1^	[67]
TROVA	Serum, urine	HPLC-FLD, column: for serum C18 Nucleosil 100-5 (125 mm × 4 mm, 5 µm) and for urine C18 Nucleosil SA (125 × 4 mm, 5 µm), mobile phase: for serum ACN and 14.3 mM tetrabutylammonium hydrogensulfate: ACN—concentrarted phosphoric acid: water (700:250:1.5:48.5 v/v) pH 3.68, for urine: acetonitrile: sodium phosphate solution (622:378) pH 3.60 isocratic elution	Protein precipitation with a mixture of ACN and HClO_4_ (99.75: 0.25) for serum, Dilution with sodium phosphate solution pH 3.6 for urine	LOD-0.02 mg L^−1^ (serum), 0.1 mg L^−1^ (urine), LLOQ—0.07 mg L^−1^ (serum), 0.5 mg L^−1^ (urine)	[86]
TROVA	Serum, urine	Differential-pulse adsorptive stripping voltammetry	Filtration	LOD—0.6 ng mL^−1^, LOQ—2 ng mL^−1^	[68]
SITA	Urine	HPLC–MS/MS, column: C18 Agilent Proshell 120-SB (50 mm × 2.1 mm, 2.7 µm), mobile phase: methanol: 0.1% formic acid (38:62 v/v), isocratic elution, temperature 40 °C	Protein precipitation in 0.1% formic acid methanol solution	LLOQ—0.025 μg mL^−1^	[87]
SITA	Plasma	HPLC–MS/MS, column: C18 ZORBAX SB (100 mm × 2.1 mm, 3.5 µm), mobile phase: methanol: 0.1% formic acid (46:54 v/v), isocratic elution, temperature 35 °C	Protein precipitation with isopropanol	LLOQ—5 ng mL^−1^	[69]
PRULI	Tablets	HPLC–UV, column: C18 Sunfire (250 mm × 4.6 mm, 5 µm), mobile phase: ACN: KH_2_PO_4_ buffer pH 7.30 adjusted with TEA (10:90 v/v), isocratic elution	Tablets were pulverized and dissolved in mobile phase	LOD—0.1404 μg mL^−1^, LOQ—0.4255 μg mL^−1^	[70]
PRULI (ULI)	Tablets	HPLC–UV, column: LUNA HILIC (250 mm × 4.6 mm, 5 µm), mobile phase: ACN: ammonium acetate (5 mM, pH 5.8) (88:12 v/v), isocratic elution	Tablets were pulverized and dissolved in methanol	LOD—0.15 μg mL^−1^ (PRULI), 3.0 μg mL^−1^ (ULI) LLOQ—0.25 μg mL^−1^ (PRULI), 5.0 μg mL^−1^ (ULI)	[88]
PRULI (ULI)	Aqueous human humor	HPLC–UV, column: C8 ZORBAX ECLIPSE XDB (150 mm × 4.6 mm, 5 µm), mobile phase: ACN and 85% aqueous phosphoric acid (15:85 v/v), isocratic elution	Dilution with mobile phase	LOD—5 ng mL^−1^ (ULI), LLOQ—6 ng mL^−1^ (ULI)	[89]
PRULI	Degradation products	HPLC MS/MS, column: C18 Waters symmetry (250 mm × 4.6 mm, 5 µm), mobile phase: A: 0.2% formic acid, B: ACN, C: methanol, gradient elution	The samples of acid and basic hydrolysis were neutrilised with NaOH and HCl solutions respectively and diluted 10 times	LOD—0.02 ng mL^−1^, LOQ—0.06 ng mL^−1^	[90]
PRULI (ULI)	Plasma	HPLC–MS/MS, column: C18 Diamonsil (200 mm × 4.6 mm, 5 µm), mobile phase: methanol:water:formic acid (70:30:0.2), isocratic elution	Protein precipitation with methanol	LLOQ—0.025 μg mL^−1^ (ULI)	[71]
PRULI	Urine	CE with chemiluminescence, capillary: fused-silica capillary (47.5 cm × 75 μm i.d.), buffer: 20 mM sodium citrate, 4 mM citric acid, 10 mM sodium sulfite at pH 6.1	Dilution with water	LOD—0.084 μg mL^−1^	[91]
GEMI	Dried blood spots	HPLC-FLD, column: ZIC-HILIC (100 mm × 4.6 mm, 5 µm), mobile phase: ACN and 10 mM ammonium acetate pH 3.5 (80:20, v/v), isocratic elution	Bried blood spots were extracted with methanol	LLOD—10 ng mL^−1^, LLOQ—25 ng mL^−1^	[92]
GEMI	Plasma	HPLC-FLD, column: C18 LC-18 symmetry column (150 mm × 3.9 mm, 5 µm), mobile phase: 0.1% TFA: ACN (80:20, v/v), isocratic elution	Ultrafiltration	LLOD—10 ng mL^−1^ LLOQ—25 ng mL^−1^	[72]
GEMI	Plasma	HPLC–UV column: C18 Eurosphere-100 (250 mm × 4.6 mm, 5 µm), mobile phase: methanol: 1% sodium acetate: orthophosphoric acid (65:35:0.5 v/v/v) pH 2.1, isocratic elution	LLE with chloroform and acetic acid mixture (5.4:0.1, v/v)	LLOQ—0.3 μg mL^−1^	[93]
GEMI	Plasma	HPTLC—10 cm × 20 cm plates 60F_254_ precoated with silica gel, mobile phase: ethyl acetate:methanol:ammonia (8.0:4.0:3.0, v/v/v), detection wavelength 254 nm	LLE with chloroform and acetic acid mixture (5.9:0.1, v/v)	LLOQ—0.5 μg mL^−1^	[93]
GEMI	Reaction mixture	HPLC—PDA column: C18 Intersil-ODS-3 V (250 mm × 4.6 mm, 5 µm), mobile phase: 0.1% TFA (pH 2.5): methanol, gradient elution, temperature 27 °C	The reaction mixture was neutralized and diluted	LOD—0.1 μg mL^−1^, LOQ—1.0 μg mL^−1^	[94]
GATI	Urine, blood	CE with electroluminescence, capillary: fused silica capillary (40 cm × 50 μm i.d.) 12 kV), running buffer: 10 mM PBS pH 5.0	SPE	LOD—0.2 ng mL^−1^, LOQ—0.5 ng mL^−1^	[73]
GATI	Food samples (muscle, liver and kidney)	HPLC–UV, column: C18 Zorbax SB-Aq (250 mm × 4.6 mm, 5 µm), mobile phase: A: methanol, B: ACN C: 0.02 M citric acid and 0.03 M ammonium acetate, temperature 35 °C, gradient elution	Accelerated solvent extraction (ASE) with ACN	LOD—3 μg kg^−1^, LOQ—10 μg kg^−1^	[96]
GATI	Food samples (muscle, liver and kidney)	HPLC MS/MS, column: Hypersil Golden (150 mm × 2.1 mm, 3.5 µm), mobile phase: A: methanol, B: ACN, C: 5 mM ammonium acetate and 0.2% formic acid, gradient elution	Accelerated solvent extraction (ASE) with ACN	LOD—0,3 μg kg^−1^, LOQ—1.0 μg kg^−1^	[96]
GATI	Plasma	HPLC–UV, column: C18 Xterra MS (50 mm × 3.0 mm, 5 µm), mobile phase 0.025 M Na_2_HPO_4_ (pH 3.0): ACN (80:20 v/v), isocratic elution	Ultrafiltration	LOQ—0.1 μg mL^−1^	[97]
GATI	Pharmaceutical formulations	HPLC–UV, column: C-18-DB SUPELCO 516 (250 mm × 4.6 mm, 5 µm), Na_2_HPO_4_ (pH 3.3): ACN (75:25 v/v), isocratic elution	Extraction from pharmaceutical formulation	LOD—0.507 μg mL^−1^, LOQ—1.538 μg mL^−1^	[98]
GATI	Tablets	HPLC–UV, column: C18 HiQ Sil (250 mm × 4.6 mm, 5 µm), mobile phase 0.01 M KH_2_PO_4_ (pH 3.0): ACN (70:30 v/v), isocratic elution	Tablets were powdered and diluted with mobile phase	LOD—0.3 μg mL^−1^, LOQ—0.5 μg mL^−1^	[99]
GATI	Plasma	HPLC-FLD, column: C18 Shim-Pack CLC-ODS (250 mm × 4.6 mm, 5 µm), mobile phase: 2.5 mM phosphoric acid: methanol: ACN: TEA pH 2.8 (64.8:15:20:0.2), isocratic elution, temperature 28 °C	SPE	LLOQ—20 ng mL^−1^	[95]

*ACN* acetonitrile, *ALOD* absolute LOD, *ASE* accelerated solvent extraction, *BALO* balofloxacin, *ALOQ* absolute LOQ, *BAL* bronchoalveolar lavage, *BCE* background electrolyte, *BCG* bromocresol green, *BPB* bromophenol blue, *CE* capillary electrophoresis, *CMPA* chiral mobile phase additive, *DAD* diode array detector, *FLD* fluorescent detector, *GATI* gatifloxacin, *GEMI* gemifloxacin, *HPLC* high performance liquid chromatography, *HPTLC* high performance thin layer chromatography, *HILIC* hydrophilic interaction liquid chromatography, *LEVO* levofloxacin, *LLE* liquid–liquid extraction, *MBTH*, 3-methyl-2-benzothiazolinone hydrazone hydrochloride, *MEPS* microextraction by packed sorbent, *MOXI* moxifloxacin, *MS* mass spectrometry, *NMR* nuclear magnetic resonance, *PAZU* pazufloxacin, *PDA* photodiode array, *PRULI* prulifloxacin, *SITA* sitafloxacin, *SPAR* sparfloxacin, *TBAA* tetrabutylammonium acetate, *TBA*·*Cl*, tetrabutylammonium chloride, *TBAmBR* tetrabutylammonium bromide, *TEA* triethylamine, *TFA* trifluoroacetic acid, *TROVA* trovafloxacin, *ULI* ulifloxacin, *UPLC* ultra performance liquid chromatography, *UV* ultraviolet

The pre-dominant type of chromatographic column used for RP-HPLC analysis is C18; however, there are other columns, e.g. C8 or C4, on which the separation is performed (Table 1). Watabe et al. tested different types of columns, e.g. C18 and C8, in LEVO and also pazufloxacin (PAZU) analysis. It was mentioned that LEVO and PAZU interact better with the C8 column because this column possesses less steric hindrance than the C18 column. The structure of these substances differs in the C-10 position of 7-oxopyrido[1,2,3-de] [1, 4] -benzoxazine-6-carboxylic acid. LEVO and PAZU posses a 4-methylpiperazinyl group and 1-aminocyclopropyl group, respectively. The presence of these groups may cause a better interaction with the surface of the stationary phase. Fang et al. used the C4 column in the separation. In this analysis, besides LEVO, also isoniasid and rifampicine were detected. The analysed compounds were in a wide range of polarity, and this type of column was more suitable than C18. The butyl bonded stationary phase provides a shorter time of analysis of non-polar compounds without significantly affecting the separation of the polar ones. The high resolution is still maintained when compared with a long chain bonded stationary phase. HILIC columns were also applied in LEVO analysis. The main advantage to using HILIC columns is the fact that they can be used for separation of ionized compounds. The HILIC columns are suitable for MS detection due to the high content of organic solvent. HILIC separation is a normal type of separation, but the typical reversed phase eluents are used. It is helpful when the poor retention of the analyte is observed in the column [26, 32, 34]. Methanol is often used in addition to ACN, and it can be used in both isocratic [23] and gradient elution [19, 23, 27, 28]. ACN, water, and methanol (and their mixtures) might be used as the solvents for stock solutions [37]. The other contents of the mobile phase might be chiral mobile phase additive (CMPA) solution consisting of CuSO_4_ and l-leucine [18], formic acid (in MS detection) [33, 35], sodium dodecylosulfate (SDS) [20], tetrabutylammonium acetate (TBAA) [20], citric acid [20, 22], ammonium acetate [22, 35], tetrabutylammonium bromide (TBAmBr) [29], and l-isoleucine [23]. Liang et al. [20] reported the use of SDS in mobile phase as an agent that increases the retention time not only for LEVO, but also for gatifloxacin (GATI), moxifloxacin (MOXI), and trovafloxacin (TROVA). It was used in addition to 25 mM phosphate buffer and ion pair reagent (10 mM TBAA), which improved the shape of the peaks. This composition of the mobile phase makes it possible to overcome the secondary interactions between silanol groups on the stationary phase and amino groups on quinolones. The addition of CuSO_4_, l-leucine or l-isoleucine enables the stereospecific determination of LEVO in matrix. Stereoselectivity was achieved through incorporation of chiral ligand exchange reagents directly into mobile phase. The Cu^2+^ ions, l-leucine, and water form a complex that combines with LEVO and its R-enantiomer. These complexes have different configurations. They might be applied for the determination of impurities in pharmaceutical formulations. The other aminoacids were tested (l-phenylalanine, l-serine and l-alanine); however, the best resolution was observed for l-leucine [18]. Devi et al. reported also the method for determination of impurities after oxidative degradation; however, it was not stereospecific [19].

The next separation technique that might be applied for LEVO analysis is CE. This method requires a relatively small amount of analyte. It may be applied for quantification of LEVO in different matrices such as human urine, tablets, or in water. The separation might be performed in both aqueous [38, 39] and in nonaqueous [40] conditions. The optimum pH of the aqueous solution is about 8.0. The change in pH may influence the response of the detector and it may cause the interaction with capillary wall for pH lower than 2.5. In comparison with chromatographic methods the CE separation is more complicated because there are more factors that influence the resolution of the analysis (pH, voltage, temperature, length of the capillary). The impurities in the sample may absorb in the wall of capillary, thus prolonging the time of the analysis.

The other technique that is applied in LEVO analysis is UV–Vis spectroscopy. This method is suitable for analysis of pure substances and pharmaceutical formulation. LEVO might be detected as the complex with bromophenol blue (BPB) or bromocresol green (BCG) [41] or as itself [42]. Spectroscopy be applied to analyse marketed formulations, as well as for human urine or serum. In this case a fluorescence detector is applied, and the fluorescence is enhanced by SDS micelle [43]. In addition to the UV–Vis spectroscopy, also ^1^H NMR, adsorptive square-wave anodic stripping voltammetry and synchronous scanning room temperature phosphorimetry may be applied [44–46]. These techniques are rarely used, and they are suitable for analysis of pharmaceutical formulation.

All aforementioned techniques require proper sample preconditioning. The following matrices may be used: serum [26, 43], plasma [20, 21, 23, 24, 27, 28, 31, 32, 34, 45], blood [30], urine [23, 38, 43–45], sputum [36], tissues [33–35], bone [21], bronchoalveolar lavage (BAL) [21], bile [30], water [40], dialysate [28], reaction mixture [19], and pharmaceutical formulation [18, 22, 25, 39, 41–43, 45, 46]. For protein precipitation, an extraction step, dilution is typically applied prior to analysis. Protein precipitation is mainly performed using solvents such as ACN [27, 31, 38], methanol [34], a mixture of ACN and methanol [24], perchloric acid and methanol [26], and trifluoroacetic acid (TFA) [28]. Watabe et al. reported the mixture of methanol and 6% perchloric acid as a precipitating agent. The use of ACN or ethanol resulted in broad or very small peaks; using methanol the supernatant was not clear enough. The use of pure 6% perchloric acid resulted in low recovery. A mixture of 6% perchloric acid and methanol resulted in good recovery of the drug. It was considered that adding methanol to the solution of perchloric acid causes co-precipitated drug with the protein to be extracted into the supernatant [26]. The extraction method comprises liquid–liquid extraction (LLE) with dichloromethane [23, 32], chloroform [41], hexane [35], solid-phase extraction (SPE) [21, 33], extraction on pre-column [29], dispersive liquid–liquid microextraction [40], and boiling in the Soxhlet apparatus [46]. Other procedures applied in preconditioning are microdialysis [30], ultrafiltration [20], microextraction [36], and dilution [19, 22, 25, 39, 42–45]. Liang et al. [20] used a mixture of SDS with ACN in ultrafiltration method as a displacing reagent in the sample preconditioning in analysis of LEVO, MOXI, GATI, and TROVA to displace the drug bound with the protein and to determine the total drug concentration which resulted in recovery greater than 95%. Xu et al. [35] tested different conditions of LLE involving a ACN/water-based solution with hexane. The non-hexane layer contained ACN, water, and an addition of phosphoric or formic acid in different proportions. For the purpose of the method the most optimal proportions of the mixture was ACN containing 0.3% phosphoric acid (70:30). The mixture of ACN and aqueous solution was used in order to prevent the sample solidification when pure ACN is used in sample preconditioning. On the other hand too low content of ACN results in not complete protein precipitation. The abovementioned proportion resulted in optimum recovery and removal of the protein.

The extraction or precipitation techniques are applied mainly in biological matrices such as plasma, serum, tissue homogenate, BAL, and urine. The dilution can be found often in sample preconditioning of pharmaceutical formulations. The analysis of the levels of LEVO with separation techniques requires also the use of the internal standard. The addition of the internal standard provide the repeatability of the results and improves the precision of the assay.

The limit of detection (LOD) and limit of quantification (LOQ) strongly depended on both the used matrix and applied detector. The LOD and LOQ for pharmaceutical formulations were even of the order of 10^−9^ g mL^−1^ for fluorescent detection. The detection limit for the biological matrices such as plasma, urine, and serum were higher. The MS detector was more sensitive for the analyte than UV or FLD; however, for routine clinical practice it is not always necessary to detect very low concentrations because peak and trough concentrations are on the order of mg L^−1^ [47].

### Balofloxacin

To determine BALO, the HPLC technique with MS and UV detection was used [15, 48]. The separation was performed on a C18 column. The organic eluents were ACN and methanol. The inorganic contents were aqueous solutions of ammonium acetate [15] and potassium dihydrogen phosphate [48]. The pH of the mobile phase was slightly acidic [15, 48] (Table 1). The selection of dihydrogen phosphate adjusted to pH 6.5 resulted in good resolution of the analysis and reduction of the tailing [48]. On the other hand Bian et al. [15] tested 10 mM ammonium acetate in different pH conditions (6.65 vs 3.0). The application of the solution with lower pH resulted in better resolution of the analysis and reduction of the tailing of the peak. These differences might be caused by the use of the different organic solvents—ACN and methanol in [48] and [15], respectively. The sample preparation involved LLE for plasma [15] and dilution for pharmaceutical formulation [48]. In LLE a mixture of dichloromethane-ethyl acetate was used. In comparison with the mixture *n*-hexane–isopropanol it showed high efficiency and less interference. During the extraction procedure, it was not advisable to use an acid (1 M HCl) or a base (1 M NaOH) because this resulted in higher interference [15]. The LOD and LOQ were lower for MS detection [15]. The quasimolecular ion [M + H]^+^ of *m*/*z* 390 of BALO was selected for monitoring [15].

### Pazufloxacin

The methods for determination of PAZU comprise both HPLC [26, 27, 49] and CE technique [50–52]. The analysed matrices were pharmaceutical formulation (tablets), milk [50], and biological fluids, i.e., serum, plasma, urine, muscle homogenate, saliva, gingiva, and crevicular fluid [26, 27, 49, 51, 52]. The applied columns in HPLC were C18 [27, 49] and C8 [26]. The content of ACN in the mobile phase did not exceed 15.5% [26, 49]. The other contents were 0.5% phosphoric acid containing 1% of TEA [49] or 1% TEA solution [26] or 0.1% formic acid at pH 3.0 adjusted with TEA [27]. CE separation was performed at room temperature, and the background electrolyte (BGE) was an aqueous solution of TRIS and phosphoric acid [50, 51]. The other additives were phosphates and β-cyclodextrines [52]. The pH was within the range 5.04–9.00. The length of capillary depended on applied voltage. A lower voltage was applied and a shorter capillary was used (Table 1). The sample preparation involved protein precipitation with methanol [49], ACN [27] or 6% perchloric acid and methanol [26], LLE with dichloromethane [50, 51], and dilution [52]. The LOD depended on a sample preparation—for extraction it was within the range 0.01–0.3 μg mL^−1^ [49–51], for protein precipitation 0.01–0.1 μg mL^−1^ [26, 27], and for diluted urine it was the highest at 7 μg mL^−1^ [52] (Table 1).

### Sparfloxacin

The method for SPAR analysis comprises spectroscopic and chromatographic techniques. The separation techniques comprise HPLC [35, 53–55], ultra performance liquid chromatography (UPLC) [17], and CE [56]. Gupta et al. [17] compared HPLC with UPLC. It was found that the tailing effect is reduced and the elution time of SPAR was 10-fold lower in UPLC analysis. The number of theoretical plates was three times greater for UPLC than for HPLC The detection applied in chromatographic analysis were UV [17, 53, 54], diode array detector (DAD) [57], and MS [35, 55]. The MRM employed in MS/MS analysis were: *m*/*z* 393.2 → 349.3 [35] and *m*/*z* 392.9 → 348.7 [55]. The separation was performed on a C18 column. The analysed matrices via separation techniques were plasma, urine, serum, and tissues (muscle) [35, 53–57]. The organic solvent applied in analysis was ACN [17, 35, 53–55, 57]. In isocratic elution the content of ACN was up to 80% [55]; however, in other analyses the content was within the range 12–20% [53, 54]. In addition to ACN, methanol was also used [53]. The other additives in the mobile phase were 0.1% orthophosphoric acid [17], 5% acetic acid [53], NaH_2_PO_4_ [54], 54 mM formic acid [35], 10 mM CH_3_COONH_4_ [35, 55], and 0.1% TFA [57]. The addition of acid resulted in slight acidic pH of the mobile phase. The ionic pair reagent was found in [53] and the content was 1% in a mobile phase. In three methods the higher temperature was reported: 30 °C [35], 35 °C [54], and 50 °C [53].

CE was performed in tetraborate buffer with an addition of silica nanoparticles. The pH was about 9.0 [56].

The sample pre-conditioning involved protein precipitation with 20% HClO_4_ [53] and ACN [55, 57], LLE with ethyl acetate [54], extraction with ACN/water mixture with addition of phosphoric acid and hexane in muscle tissue [35], and dilution [17, 53, 56]. The addition of a small amount of phosphoric acid increased the recovery of SPAR from the muscle tissue. The authors also used the formic acid; however, the recovery in this case was lower [35]. The detection limits strongly depended on the applied detection. The lowest were noted for MS detection [55] (Table 1).

## Fourth Generation Fluoroquinolones 

The representatives of fourth generation fluoroquinolones are MOXI, TROVA, sitafloxacin (SITA), prulifloxacin (PRULI), gemifloxacin (GEMI), GATI [12]. MOXI is characterized by a wide range of activity. The activity comprises Gram-negative and Gram-positive bacteria, such as *Staphylococcus*, *Streptococcus*, *Enterococcus*, and also atypical bacteria and anaerobes [58–61]. It is used in the treatment of conjunctivitis, keratitis, pre- and postoperatively to control infections of the eyes. LEVO is used in CAP and in multidrug resistant tuberculosis (MDR-TB) treatment [62]. The killing effect on slow replicating bacilli in the tissues is an important factor that shortens MDR-TB treatment and, therefore, MOXI is often added to standard therapy [63–66]. TROVA exhibits a broad activity spectrum against Gram-positive and Gram-negative bacteria. It is used mainly in veterinary medicine—it was withdrawn from the market in 1999 due to incidents of idiosyncratic hepatotoxicity [67, 68]. SITA is active against Gram-positive and Gram-negative bacteria, *Chlamydia* spp., and *Mycoplasma* spp. It also shows activity against quinolone-resistant methicilin-resistant *S. aureus*, *Pneumococcus* spp., and *Pseudomonas* spp. [69]. PRULI is a prodrug of ulifloxacin (ULI). PRULI is rapidly metabolized by paraoxogenases to ULI. It is applied in simple cystitis treatment, acute exacerbation of chronic bronchitis, and lower urinary tract infections in children and adults [70, 71]. GEMI is a broad bactericidal spectrum drug. It has particularly enhanced activity against Gram-positive organisms. It also shows fourfold higher bactericidal activity against *S. pneumoniae* than MOXI and is active against *H. influenzae* and *M. catarrhalis* and the atypical organisms *L. pneumophila*, *Chlamydia* spp., *Mycoplasma* spp. It is also applied in urinary tract infections [72]. GATI is active against Gram-positive and Gram-negative bacteria. It is active in vitro against clinically important pathogens such as penicillin-resistant *S. pneumoniae* [73].

### Moxifloxacin

MOXI may be analysed by many analytical techniques. The predominant ones are reversed phase HPLC with different detectors such as UV [20, 24, 54, 60, 74, 75], FLD [27, 29, 76–79], MS [80–82], and DAD [57, 66]. There are also reported methods for CE [32, 83, 84]. A simple method involving UV–Vis spectroscopy is applied in analysis of pharmaceutical formulation and pure substance [85].

The main constituent of the mobile phase in HPLC separation was the phosphate buffer [24, 29, 54, 66, 74–76, 78]. The concentration of the phosphate buffer was within the range 10–50 mM; however, there is also a reported method with a high concentration of sodium phosphate (0.25 M) [78]. The other constituent of the aqueous phase might be carboxylic acid such as citric acid [20], formic acid [27], acetic acid [81], TFA [77] or its anhydride [81]; organic salts such as ammonium acetate [81] and SDS [20]. Chan et al. [77] reported the use of TFA because it doesn’t affect the fluorescence signals. In many methods an ion pair reagent was the constituent of the mobile phase. The most common used was TEA [60, 66, 74, 75, 80]. The concentration was in the range 0.03–2.00%. The lowest concentration was applied in the method with MS-detection [80]. For the other detectors the minimum concentration of TEA was 0.1%. The other ion pair reagents were TBAA [20], TBAmBr [29], and tetrabutylammonium chloride (TBA·Cl) [77, 79]. The ion pair reagent reduces the tailing of the peaks due to the interaction with the silanol groups. It reduces both the availability of free stationary phase silanols and the analyte’s interaction with them. The addition of ion pair reagent should be as low as possible. The high content causes a long column equilibrating time, and it is difficult to wash off the column. The high tailing is also observed for pH 4.5 and 5.5. The negatively charged silanol groups from the stationary phase and positively charged amine group of MOXI are responsible for it. The decrease of pH value to 3.5 results in reducing of peaks tailing. The silanol groups above pH 3.5 are ionized and interact with 1° and 2° amines [74]. The content of the water-based phase in isocratic elution was 57–95%. The organic solvents applied in the chromatographic analysis were ACN [20, 24, 29, 54, 57, 60, 76, 78, 79, 81, 82], methanol [66, 74, 80], or both [27, 75, 77]. The pH of the mobile phase was acidic 2.5–6.0. Laban-Djurdević et al. [78] optimized the condition of the analysis by the response surface method in two factor space. The statistical analysis was performed by the Statistica v.6 software and it occurred that the most important factors influencing retention time and resolution were ACN content and pH of the mobile phase. Less significant occurred to be the ionic strength of the phosphate buffer. It was observed that response surface possess a relatively flat maximum situated between 10 and 15% ACN and pH within the range 3.0–4.5.

In MS/MS analysis the following MRM transitions were applied: *m*/*z* 402.1 → 260.0 [80], *m*/*z* 402.0 → 358.2 [81], and *m*/*z* 402 → 384, *m*/*z* 402 → 358 [82].

The next separation technique applied in MOXI analysis is CE. The BGE in reported method consisted of buffers (both organic and inorganic), salts, and TEA [39, 83, 84]. In order to improve the resolution for enantiomers the sulfated γ-cyclodextrin were applied [84]. The pH depended of the constituents of the mobile phase and it was both acidic or base. The measurements were performed at the room temperature.

Ashour et al. reported the simple technique for analysis of MOXI in both pharmaceutical formulation and pure substance [85]. The method was based on the coupling reaction of MOXI with 3-methyl-2-benzothiazolinone hydrazone hydrochloride monohydrate (MBTH) in the presence of Ce(IV) ions. This method is suitable for kinetic measurements. Djurdevic et al. [60] and Cruz et al. [84] reported the methods that were also suitable for impurities analysis. They are based on HPLC and CE. The HPLC method was suitable for analysis of impurities and forced degradation products [60]. The CE method was suitable for determination of S,S-, R,R-, R,S-, and S,R-diastereoisomers of MOXI. The active one was S,S-isomer, the other ones were potential chiral degradation products of MOXI [84].

The preconditioning of the sample involves the following techniques such as extraction [29, 54, 75, 79, 81–83], protein precipitation [24, 27, 57, 76, 80], dilution [39, 60, 66, 74, 77, 79, 84, 85], and filtration [20, 78]. The extraction techniques comprise classical LLE with dichloromethane [75, 83], ethyl acetate [54], SPE [79, 82], and others such as online extraction on pre-column [29] and extraction with cyanoimipramine [81]. The protein precipitation was performed by ACN [27, 57, 77], methanol [80], mixture of ACN and methanol [24], and HClO_4_ [76].

The analysed matrices were plasma [20, 24, 27, 54, 57, 75, 78, 80, 82], serum [29], blood [81], saliva [76], muscle [83], vitreous and aqueous humor [77, 79], and pharmaceutical formulations (eye drops, tablets), as well as in pure substance [39, 60, 66, 74, 84, 85]. The detection of MOXI in biological matrices involved mainly extraction or protein precipitation step. However, ultrafiltration with SDS as an displacing agent or microfiltration was also applied. The displacing reagent was used as a drug releasing factor from the proteins. It enhances the protein solubility and minimizes binding with the drug. The different concentrations of SDS were tested, and it was found that the most optimum was 10 mM SDS and phosphoric buffer adjusted to pH 3.0. Addition of SDS also increased the fluorescence intensity [78]. The preconditioning of the sample in pharmaceutical formulation was dilution. Internal standards were used in the most of the methods.

The LOD and LOQ mainly were of the order of μg mL^−1^. However, both MS and FLD or UV detection enabled to detect the levels of ng mL^−1^ (Table 1).

### Trovafloxacin

TROVA is analysed by HPLC with both UV and FLD [20, 27, 67, 86]. The separation is performed on a C18 column. The organic solvent applied in the analysis was ACN [20, 86] or as a mixture with methanol [27, 67]. Ion-pair reagent used were TBA as hydroxide [67], hydrogensulfate [86], or acetate [20] and TEA [27]. The inorganic content was dihydrogenphosphate sodium [67], sodium phosphate [86], or 0.1% formic acid [27]. The other substances found in the mobile phase were SDS and citric acid [20]. The pH of the mobile phase was slightly acidic. The analysed matrices were plasma and urine. The sample preconditioning was protein precipitation (for plasma or serum) with ACN [27], a mixture of ACN and perchloric acid [86], and 20% perchloric acid [67]. The other technique was ultrafiltration with 0.5% SDS solution preconditioning [20]. Urine was diluted prior to analysis [86]. The LODs were similar for the mentioned chromatographic methods with protein precipitation. The detection limit was higher for urine. TROVA was also analysed by differential-pulse adsorptive stripping voltammetry [68]. In this case the sample preconditioning was filtration and LOD was higher than for the formerly reported methods (Table 1).

### Sitafloxacin

SITA was analysed by chromatographic methods with the mass detection [69, 87]. The MS/MS analysis was based on the following MRM transitions: *m*/*z* 410.1 → 392.1 [87] and *m*/*z* 410.1 → 392.2 [69]. The composition of the applied mobile phase in reported methods was similar—it was a mixture of methanol and 0.1% formic acid. In the method where the content of formic acid solution was higher (62 vs. 54%) the temperature of the separation was also higher, i.e. 40 vs. 35 °C. In both cases the protein precipitation was the method for sample preconditioning. The precipitating agent was methanol with a 0.1% addition of formic acid [87] or isopropanol [69]. The quantitation limit was lower in case of [87] (Table 1).

### Prulifloxacin

PRULI is a prodrug of ULI. Some reported methods for PRULI determination also reports the determination of ULI. ULI was also considered as an impurity of PRULI [88]. Both compounds were analysed by HPLC techniques with UV [70, 88, 89] or MS detection [71, 90] and CE [91]. The following MRM transitions were observed for PRULI in MS/MS analysis: *m*/*z* 462 → 444; *m*/*z* 462 → 418; *m*/*z* 460 → 360; *m*/*z* 462 → 350 [90]. These transitions are helpful in analysis of degradation products of PRULI. For ULI the following MRM in MS/MS detection was employed *m*/*z* 350 → 248 [71]. The analysed matrices were tablets [70, 88], degradation products [90], aqueous human humor [89], plasma [71], and urine [91]. The separation was performed on C18, C8, and HILIC columns. The mobile phase consisted of ACN [70, 88–90] and methanol [71] as an organic content. ACN can be replaced with alcohol; however, in this case its content must be higher to achieve the same degree of retention on HILIC column [88]. The other contents of mobile phase were dihydrogen potassium phosphate [70], ammonium acetate [88], phosphoric acid [89], formic acid in MS detection [71, 90]. The BGE in CE consisted of sodium citrate, citric acid, and sodium sulfite [91]. The sample preconditioning involved pulverization and dissolution with a proper solvent in case of tablets [70, 88], dilution in case of aqueous matrices [89–91], protein precipitation with methanol [71]. The limits of detection depended on the applied matrix and the detector. The lowest were for the MS detection for PRULI [90]. The same type of detection for ULI resulted in higher LOD [71]. In the methods where PRULI and ULI were detected simultaneously the detection limits were lower for PRULI [88] (Table 1).

### Gemifloxacin

GEMI was analysed by HPLC and high performance thin layer chromatography (HPTLC). The HPLC methods involved FLD, UV, and PDA detection [72, 92–94]. The HPTLC plates were detected in UV light [93]. The applied columns were C18 [72, 93, 94] and HILIC [92]. As in the previously reported method the applied organic solvents were ACN [72, 92], methanol [93, 94]. The aqueous solutions contained 10 mM ammonium acetate [92], 0.1% TFA [72], sodium acetate [93], orthophosphorc acid [93]. Ethyl acetate and ammonia were used in HPTLC separation [93]. The analysed matrices were dried blood [92], plasma [72, 93], and reaction mixture [94]. The sample preconditioning involved extraction with methanol for blood spots [92], ultrafiltration [72], LLE [93], and dilution [94]. The quantification limits were lower for FLD detection [72, 92] than for UV and PDA detection [93, 94] (Table 1).

### Gatifloxacin

In GATI analysis were applied HPLC and CE. The HPLC techniques involved FLD [20, 27, 29, 79, 95], UV detection [20, 24, 25, 54, 96–99], and MS detection [96]. The detection in CE involved UV detection [39, 52, 56], capacitively coupled contactless conductivity detection [83] and electroluminescence [73].

The chromatographic analysis was performed on C18 column. The organic eluent was ACN [20, 24, 25, 27, 29, 54, 79, 95–97], methanol [27, 95, 96]. Ion pair reagents were TBAA [20], TEA [25, 27, 95], TBAmBr [29], TBA·Cl [79]. The other constituents were phosphates [24, 29, 54, 97–99], SDS [20], citric acid [20, 96], 0.1% FA [27, 96], TFA [79], ammonium acetate [96], phosphoric acid [95]. The pH of the mobile phase was acidic. The elution was both iscocratic [20, 24, 29, 54, 79, 95, 97–99] and gradient [27, 96]. HPLC analysis was performed at ambient temperature with the exception of [54, 96] and [95], where it was performed at 35 and 28 °C, respectively. CE separation was performed on PBS [73], TRIS/hydrochloride [39], tetraborate buffer [39], phosphate buffer with cyclodextrins [52], disodium tetraborate with silica nanoparticles [56], tartaric acid, and sodium acetate [83]. The following MRM transitions were employed in MS/MS analysis: *m*/*z* 375.9 → 332.0 and *m*/*z* 375.9 → 260.9 [96].

The analysed matrices were tablets [39], pharmaceutical formulations [25, 98], serum [29, 56], plasma [20, 24, 27, 53, 54, 95, 97], blood [73], urine [52, 53, 73], muscle [83], aqueous and vitreous humor [79], and food samples [96].

The sample preconditioning involved pulverization and dissolution [25, 39, 99], ultrafiltration [20, 97], online extraction on column [29], protein precipitation with ACN [27] or methanol or a mixture of ACN and methanol [24], dilution with a proper solvent [52, 56, 79], LLE [54, 83, 98], SPE [73, 79, 95], and ASE (accelerated solvent extraction) [96]. Fu et al. [73] reported that SPE lead to successful clean-up of the sample and that no unacceptable interference was observed during the analysis. Tasso et al. [95] reported the method of online SPE combined with HPLC. The biological sample was injected onto a cartridge and it was eluted cleaned up using proper solvents and washed off on a column. The detection and quantification limits depended on the applied detection and there were lower for MS detection (Table 1).

## Conclusion

This paper presents information about methods involving different analytical and separation techniques and, therefore, might be helpful in the selection of procedures for the levels determination of the antimicrobial agent.

The fluoroquinolones posses two groups that can interact with protons—amine and the carboxyl groups. They are strongly ionized compounds due to their zwitterionic nature. It may cause that analysis of these substances with separation techniques becomes complicated. The tailing of peaks and also poor resolution on the column are the main problems that can be encountered during analysis. The most commonly used organic solvents are ACN and methanol in gradient or isocratic elution. ACN has more elution strength than methanol and often causes peaks to appear on the chromatogram. The addition of methanol may influence the resolution between peaks. The separation of fluoroquinolones is often performed on reversed phase. The applied columns are mainly C18, C8, and C4. The other columns that may be used in analysis are HILIC. They are the alternative to RP-HPLC for separation of hydrophilic ionized solutes. In this case the content of at least 80% of organic solvent (mainly ACN) is required. This type of column is desirable for separation in MS analysis. The organic solvent evaporates easily and results in low content of aqueous solution, which is not as volatile as ACN or methanol. One of the constituents of the mobile phase applied in the analysis of the fluoroquinolones is the solution of ion-pair reagents (TEA, TBAA, SDS, or others). The content of this type of reagent results in a longer column equilibrating time and may lead to problems with column maintenance. On the other hand, ion-pair reagents cause better interaction of the analyte with the stationary phase. When considering the composition of the mobile phase, researchers should take into consideration these two facts. The ion-pair reagent should be added to the mobile phase when the addition of the buffer at the proper pH value does not suppress the peak tailing effect or does not provide a good resolution of the analytes. The applied column, the addition of other contents such as organic or inorganic salts, ion-pair reagent, and also the proper value of pH influence the shape of the peak and may have an impact on LOD and LOQ. The other things to consider are the sample preconditioning and the applied detector. In quantitative analysis the applied method must be appropriate for the predicted level of the analyte, used matrix, and the aim of the analysis. The analysis performed in biological fluid will be characterized by higher LOD or LOQ than those performed in pharmaceutical formulation or aqueous solution, and they require proper preconditioning prior to analysis. The LOD and LOQ is higher in the methods involving protein precipitation in the preconditioning step than in the methods in which the extraction step (LLE or SPE) is involved. The extraction techniques are more laborious in sample preparation, but they are useful in the detection of the lower concentration of the analyte in a sample. In the analysis of pharmaceutical formulation there is no urgent need to apply the aforementioned preconditioning steps—the dilution is sufficient. The analysis in this case may be performed by UV, Vis, and fluorescence spectroscopy. Deproteinization step by protein precipitation is the least complex procedure for sample preconditioning when the removal of the proteins is required. LLE or SPE are more complex techniques because they involve the evaporation of the solvent and dissolution of the sample. These steps are the points where possible mistakes can be made. Also, they are time consuming and more expensive because they involve the use of additional reagents and equipment. The sample preparation should be as simple as possible and fitted to the matrix. There are matrices that require more complex sample precondition like tissue homogenates. In this case not only proteins should be removed, but also lipids. Protein precipitation should be done with a solvent that prevents too much solidification which may lead to the decrease of the recovery. The analyte might be trapped on the precipitate. In this case the addition of water based solution can be applied. The other advantage of protein precipitation is that the recovery of the analyte is greater than for the extraction techniques (SPE and LLE). Moreover, the difference of the hydrophobicity of the analytes must be also taken into consideration during extraction procedures. The separation techniques must be applied when there is more than one analyte that can be detected under the analysis conditions. The methods that apply fluorescent detection or mass detection are characterized by lower LOD and LOQ. The combination of mass detection with extraction step results in an LOD on the order of pg mL^−1^. MS analysis requires an organic acid as a protonating agent. The most common are formic acid, acetic acid alone or in a mixture with ammonium formate or acetate. The applied MS detection was a tandem MS/MS in most cases. The analysis of the level of the antibacterial drugs is essential from the clinical point of view in order to avoid the resistance of the microorganism on therapy. In the clinical analysis, where the fast result is required to improve the treatment with antimicrobial agent, the protein precipitation combined with HPLC should be applied. However, if it is not possible to apply MS detection, FLD or UV detection with the addition of an internal standard is a suitable technique for fast and inexpensive analysis. Fluoroquinolones of the third and fourth generation are the antibacterial agents for which the concentration in blood and other fluids are on the order of mg L^−1^ and the HPLC techniques with FLD or UV detection are suitable. The use of an internal standard also compensates for the loss of analyte during the extraction step and provides repeatable results.

## Abbreviations

ACN: Acetonitrile
ASE: Accelerated solvent extraction
AUC: Area under the curve
BAL: Bronchoalveolar lavage
BALO: Balofloxacin
BCE: Background electrolyte
BCG: Bromocresol green
BPB: Bromophenol blue
C_max_: Maximum concentration
CAP: Community acquired pneumonia
CE: Capillary electrophoresis
CMPA: Chiral mobile phase additive
DAD: Diode array detector
FLD: Fluorescence detector
GATI: Gatifloxacin
GEMI: Gemifloxacin
HILIC: Hydrophilic interaction liquid chromatography
HPLC: High performance liquid chromatography
HPTLC: High performance thin layer chromatography
LEVO: Levofloxacin
LLE: Liquid–liquid extraction
MBTH: 3-Methyl-2-benzothiazolinone hydrazone hydrochloride
MDR-TB: Multidrug-resistant tuberculosis
MIC: Minimum inhibitory concentration
MOXI: Moxifloxacin
MRM: Multiple reaction mode
MS: Mass spectrometry
NMR: Nuclear magnetic resonance
PAZU: Pazufloxacin
PDA: Photodiode array
PRULI: Prulifloxacin
SITA: Sitafloxacin
SPAR: Sparfloxacin
TBAA: Tetrabutylammonium acetate
TBA·Cl: Tetrabutylammonium chloride
TBAmBR: Tetrabutylammonium bromide
TEA: Triethylamine
TFA: Trifluoroacetic acid
TROVA: Trovafloxacin
ULI: Ulifloxacin
UPLC: Ultra performance liquid chromatography
UV: Ultraviolet
